# Steroidogenic Factor-1 Regulation of Dorsomedial Ventromedial Hypothalamic Nucleus Ghrh Neuron Transmitter Marker and Estrogen Receptor Gene Expression in Male Rat

**DOI:** 10.1080/17590914.2024.2368382

**Published:** 2024-07-15

**Authors:** Subash Sapkota, Sagor C. Roy, Rami Shrestha, Karen P. Briski

**Affiliations:** School of Basic Pharmaceutical and Toxicological Sciences, College of Pharmacy, University of Louisiana Monroe, Monroe, Louisiana, USA

**Keywords:** GAD_65_, Ghrh, insulin-induced hypoglycemia, neuronal nitric oxide synthase, sex differences

## Abstract

Ventromedial hypothalamic nucleus (VMN) growth hormone-releasing hormone (Ghrh) neurotransmission shapes counterregulatory hormone secretion. Dorsomedial VMN Ghrh neurons express the metabolic-sensitive transcription factor steroidogenic factor-1/NR5A1 (SF-1). *In vivo* SF-1 gene knockdown tools were used here to address the premise that in male rats, SF-1 may regulate basal and/or hypoglycemic patterns of Ghrh, co-transmitter biosynthetic enzyme, and estrogen receptor (ER) gene expression in these neurons. Single-cell multiplex qPCR analyses showed that SF-1 regulates basal profiles of mRNAs that encode Ghrh and protein markers for neurochemicals that suppress (γ-aminobutyric acid) or enhance (nitric oxide; glutamate) counterregulation. SF-1 siRNA pretreatment respectively exacerbated or blunted hypoglycemia-associated inhibition of glutamate decarboxylase_67_ (GAD_67_/GAD1) and -_65_ (GAD_65_/GAD2) transcripts. Hypoglycemia augmented or reduced nitric oxide synthase and glutaminase mRNAs, responses that were attenuated by SF-1 gene silencing. Ghrh and Ghrh receptor transcripts were correspondingly refractory to or increased by hypoglycemia, yet SF-1 knockdown decreased both gene profiles. Hypoglycemic inhibition of ER-alpha and G protein-coupled-ER gene expression was amplified by SF-1 siRNA pretreatment, whereas as ER-beta mRNA was amplified. SF-1 knockdown decreased (corticosterone) or elevated [glucagon, growth hormone (GH)] basal counterregulatory hormone profiles, but amplified hypoglycemic hypercorticosteronemia and -glucagonemia or prevented elevated GH release. Outcomes document SF-1 control of VMN Ghrh neuron counterregulatory neurotransmitter and ER gene transcription. SF-1 likely regulates Ghrh nerve cell receptivity to estradiol and release of distinctive neurochemicals during glucose homeostasis and systemic imbalance. VMN Ghrh neurons emerge as a likely substrate for SF-1 control of glucose counterregulation in the male rat.

## Introduction

The brain maintains systemic glucose homeostasis through control of synchronized autonomic, neuroendocrine, and behavioral motor functions that govern glucose dietary intake, cellular uptake and utilization, *de novo* synthesis, and storage as glycogen. Final common control of this vital motor outflow is imposed by the hypothalamus, the hierarchic visceral motor center of the brain. The ventromedial hypothalamic nucleus (VMN), a bilateral component of the mediobasal hypothalamus, is a key sensory and integrative element of the glucose-regulatory circuitry that assimilates telencephalic, diencephalic, and brainstem loci (Chan & Sherwin, 2013; Tu et al., 2022; Watts & Donovan, 2010). The transcription factor steroidogenic factor-1 (SF-1; NR5A1) is uniquely expressed in the VMN, where it shapes establishment of VMN nerve cell population phenotypes (Davis et al., 2004; McClellan et al., 2006). SF-1 gene repression negatively affects VMN cytoarchitectural organization, culminating in thermogenic, metabolic, and reproductive dysfunction (Kim et al., 2009; 2012; Zhao et al., 2008). SF-1 expression within the boundaries of the VMN is not ubiquitous, as it occurs in the dorsomedial (VMMdm) and central (VMNc), but not ventrolateral VMN (VMNvl) (Cheung et al., 2013; Kim et al., 2019). SF-1 participation in the neural control of bodily energy and glucose homeostasis is well documented (Choi et al., 2013; Garfield et al., 2014; Kim et al., 2011; Meek et al., 2016; Xu et al., 2011; Zhang et al., 2008).

Recent studies sought to characterize SF-1-expressing VMNdm neurons by neurochemical phenotype to facilitate understanding of how regulatory effects of this transcription factor are communicated to the neural glucostatic network (Sapkota et al., 2023). The neuropeptide growth hormone-releasing hormone (Ghrh; e.g., somatocrinin) is expressed in the VMNdm (Burgunder, 1991), where it may participate in non-hypophysiotropic mechanisms that stimulate pituitary growth hormone (GH) secretion (Frohman et al., 1968). Using combinative *in situ* immunocytochemistry/laser-catapult-microdissection/single-cell multiplex qPCR methodology within the context of a validated *in vivo* experimental model for insulin-induced hypoglycemia (IIH) (Paranjape & Briski, 2005), that work showed that VMNdm Ghrh neurons express hypoglycemia-sensitive SF-1 mRNA and that Ghrh neurotransmission is critical for optimal hypoglycemic induction of counterregulatory hormone outflow. Outcomes also established the co-presence in microdissected Ghrh nerve cell samples of gene transcripts that encode enzyme markers for several characterized counterregulation-enhancing or -constraining neurotransmitters, namely the labile gas nitric oxide (NO) and the amino acids γ-aminobutyric acid (GABA) and glutamate (Glu). A correlated observation was that Ghrh neurons express Ghrh receptor (Ghrh-R), which evidently mediates neuromodulatory effects on those gene profiles. Co-transmission of neurochemicals of diverse chemical structure, spatial, and temporal profiles that impose distinctive control of counterregulatory hormone secretion supports the prospect that VMNdm Ghrh neurons supply complex, coordinated multi-modal input to the brain glucose-regulatory network. The current research investigated the hypothesis that in VMNdm Ghrh neurons in the male rat brain, SF-1 regulates expression patterns of genes that encode the neuropeptide transmitter Ghrh and protein markers for co-expressed neurochemicals. Here, adult male rats were pretreated by bilateral administration of self-delivering Accell™ SF-1 or control/scramble siRNA to the VMN before subcutaneous (*sc*) insulin or vehicle injection. Ghrh neurons were collected by laser-catapult-microdissection from the VMNdm of each subject across all treatment groups for single-cell multiplex qPCR analysis of Ghrh, neuronal nitric oxide synthase (nNOS), glutamate decarboxylase_67_ (GAD1/GAD_67_), glutamate decarboxylase_65_ (GAD1/GAD_65_), and glutaminase (GLS) transcript profiles.

The ultra-sensitive energy gauge 5’-AMP-activated protein kinase (AMPK) undergoes activation via phosphorylation in reaction to increased cellular AMP/ATP ratio (Hardie et al., 2012, 2016; Hardie & Lin, 2017). Hypothalamic AMPK supplies vital cues on cell energy stability to neural pathways that govern bodily energy balance (López, 2018; Pimentel et al., 2013; Xue & Kahn, 2006). Ventromedial hypothalamic AMPK is implicated in neural regulation of hypoglycemic patterns of counterregulatory hormone outflow (Han et al., 2005; McCrimmon et al., 2008). Brain cell glucopenia may be directly monitored by SF-1 – expressing VMN neurons as these neurons express AMPK protein, which undergoes increased phosphorylation in response to hypoglycemia (Ibrahim et al., 2020). The AMPK alpha catalytic subunit exists as two isoforms, e.g., Prkaa1/AMPK-alpha1 (AMPKα1) and Prkaa2/AMPK-alpha2 (AMPKα2). These variants are activated to a comparable extent when AMP levels rise, but each exhibits distinctive, dissimilar substrate specificity which likely results in dissimilar effects on cell function by phosphorylation of different target proteins (Woods et al., 1996). Current research investigated the hypothesis that VMNdm Ghrh neurons express transcripts that produce one or both AMPK-alpha variants and that these gene profiles are subject to regulation by SF-1 under eu- and/or hypoglycemic conditions.

Estradiol regulates glucose homeostasis through control of carbohydrate intake and metabolism, glucose tolerance, and hepatic gluconeogenesis and glycogenesis (Ahmed-Sorour & Bailey, 1980; 1981; Bailey & Ahmed-Sorour, 1980; Lenzen & Bailey, 1984; Wurtman & Baum, 1980). Estradiol governs plasma insulin and counterregulatory hormone profiles (Adams et al., 2005; Ahmed-Sorour & Bailey, 1980; Briski & Nedungadi, 2009; Faure et al., 1988; Komesaroff et al., 1988), and acts within the female rat VMN to control plasma glucose concentrations (Nedungadi & Briski, 2012). The nuclear estrogen receptors (ER)-alpha (ERα) and -beta (ERβ) regulate VMN neurotransmitter marker protein expression (Mahmood et al., 2018). Recent reports show that VMNdm Ghrh neurons express gene transcripts for ERα and ERβ proteins and the plasma membrane G protein-coupled ER-1 (GPER), and that these gene profiles are responsive to hypoglycemia (Sapkota et al., 2023). At present, there is little insight on how if or how SF-1 may modulate estradiol actions within the VMN that control glucose homeostasis. Current research examined the corollary premise that SF-1 may regulate ER variant gene expression within VMNdm Ghrh neurons in the male rat.

## Materials and Methods

### Animals

Adult male Sprague Dawley rats (250–300 g *bw*) were housed in shoe-box cages (2–3 per cage), under a 14-hr light: 10-hr dark cycle; lights on at 05.00 hr. Before experimentation was initiated, animals were acclimated on a daily basis to handling. Animals had free access to standard laboratory chow and tap water. Study protocols and procedures were performed in conformity with the NIH Guide for Care and Use of Laboratory Animals, 8th Edition, under approval by the ULM Institutional Animal Care and Use Committee, approval No. 22SEPT-KPB-01.

### Experimental Design

On Study Day 1, animals were randomly assigned to four treatment groups (*n* = 6 per treatment group). Rats were anesthetized by intraperitoneal injection of 9.0 mg ketamine/1.0 mg xylazine/0.1 mL/100g *bw* prior to bilateral intra-VMN injection (total volume: 1.0 µL; infusion rate: 3.6 µL/min; coordinates: −2.5 mm posterior to bregma, 0.6 mm lateral to midline, 9.0 mm ventral to skull surface) of SF-1 siRNA (500 pmol; Accell siRNA rat SF-1, set of 4; prod. no. EQ-100091-00-0010; Horizon Discovery, Waterbeach, UK) or scramble (SCR) siRNA (500 pmol; Accell Control Pool Non-Targeting; prod. no. D-001910-10-20; Horizon.), as described (Sapkota et al., 2023). Injections were made using a 33 gauge Neuros syringe (prod. no. 53496; Stoelting Co., Wood Dale, IL), using a Neurostar stereotactic Drill and Injection Robot (Neurostar, Tubingen, Germany). After surgery, rats were injected with ketophen [subcutaneous (*sc*); Zoetis Inc., Kalamazoo, MI] and enrofloxacin (IM; Bayer HealthCare LLC, Animal Health Division, Shawnee Mission, KS), and treated by topical application of 0.25% bupivacaine to closed incisions; animals were transferred to single-occupancy cages after full recovery from anesthesia. On Study Day 7, animals were injected *sc* at 09.00 hr with vehicle (V; sterile diluent; Eli Lilly & Co., Indianapolis, IN) or neutral protamine Hagedorn insulin (INS; 10.0 U/kg *bw* (Napit et al., 2019); Eli Lilly); animals were sacrificed by rapid decapitation one hr post-injection. Individual brains were dissected whole, then snap-frozen by immersion in liquid nitrogen-cooled isopentane for storage at −80 °C. Each brain was cut into 10 µm- or 100 µm-thick frozen sections, over alternating distances of 100 µm (1 × 100 µm sections) and 100 µm (10 × 10 µm thin sections), respectively, between −1.80 to −2.3 mm posterior to *bregma*.

### Laser-Catapult-Microdissection of VMNdm Ghrh Neurons

Individual 10 µm-thick fresh-frozen sections were mounted on polyethylene naphthalate membrane-coated slides (prod. no. 415190-9041-000; Carl Zeiss Microscopy LLC, White Plains, NY). Tissues were fixed with ice-cold acetone (5 min), then blocked with 1.5% normal goat serum (prod. no. S-2000, Vector Laboratories, Burlingame, CA) in Tris-buffered saline, pH 7.4, (TBS), 0.05% Triton X-100 (2 hr), before incubation with a rabbit primary antiserum raised against preproGhrh (prod. no. PA5-102738, 1:2000; Invitrogen, Waltham, MA) (48–72 hr; 4 °C) (Sapkota et al., 2023). Sections were next incubated with a horseradish peroxidase-labeled goat anti-rabbit secondary antibody (prod. no. PI-1000, 1:1000; Vector Lab.; 1 hr), then processed with ImmPACT 3,30-diaminobenzidine peroxidase substrate kit reagents (prod. no. SK-4105; Vector Lab.) to visualize cytoplasmic Ghrh-immunoreactivity (-ir). For each animal, individual Ghrh-ir-positive neurons were detached and propelled from tissue sections using a Zeiss P.A.L.M. UV-A microlaser IV system, as reported (Sapkota et al., 2023), and collected into an adhesive cap (prod. no. 415190-9181-000; Carl Zeiss) containing lysis buffer (4 µL; Single Shot Cell Lysis Kit, prod. no. 1725080; Bio-Rad Laboratories, Hercules, CA) for multiplex gene expression analysis.

### Single-Cell Multiplex Quantitative Reverse Transcription PCR (RT-qPCR) Analysis: Complementary DNA (cDNA) Synthesis and Amplification

Single-cell lysates were centrifuged (3000 rpm; 4 °C), then incubated in an iCyclerQ RT-PCR Detection System (Bio-Rad) at 25 °C (10 min), then 75 °C (5 min). Sample RNA integrity, purity, and quantity were determined by ThermoFisherScientific NanoDrop One^c^ microvolume UV-Vis spectrophotometry. Single-cell mRNA samples were reverse-transcribed to cDNA by addition of 1.5 µl cDNA synthesis buffer (iScript^TM^ Advanced cDNA Synthesis Kit. prod. No. 1725038; Bio-Rad) prior to initial incubation at 46 °C (20 min), followed by secondary incubation at 95 °C (1 min), as described (Ali et al., 2022a, 2022b; Alshamrani et al., 2022). A pre-amplification master mix was prepared by combining PrimePCR^™^ PreAmp for SYBR Green Assays for SF-1/NR5A1 (prod. no. qRnoCID0001458), Ghrh (prod. no. qRnoCID0007723), GAD_67_/GAD1 (prod. no. qRnoCID0004554), GAD_65_/GAD2 (pro. no. qRnoCID0003485), NOS1/nNOS (prod. no. qRnoCED0009301), GLS (prod. no. qRnoCID0007756), PRKAA1/AMPKα1 (prod. no. qRnoCID0001262), PRKAA2/AMPKα2 (prod. no. qRnoCID0006799), ESR1/ERα (prod. no. qRnoCID0009588), ESR2/ERβ (prod. no. qRnoCID0008785), GPER (prod. no. qRnoCED0007818), Ghrh-R (prod. no. qRnoCED0003825), and GAPDH (prod. no. qRnoCID0057018; Bio-Rad) with SsoAdvanced^™^ PreAmp Supermix (prod. no. 1725160; Bio-Rad). Pre-amplified cDNA was produced by the addition of 9.5 µL preamplification master mix to individual cDNA samples prior to thermal cycler incubation at 95 °C (3 min), followed by 18 cycles of incubation at 95 °C (15 sec), then 58 °C (4 min). Pre-amplified cDNA samples were diluted with IDTE (185 µL; prod. No. 11-05-01-05; 1X TE solution; Integrated DNA Technologies, Inc., Coralville, IA). *RT-qPCR Analysis:* PCR samples were prepared by combining Bio-Rad primers [SF-1/NR5A1 (0.5 µL; prod. no. qRnoCID0001458), Ghrh (0.5 µL; prod. no. qRnoCID0007723), GAD1/GAD_67_ (0.5 µL; prod. no. qRnoCID0004554), GAD2/GAD_65_ (0.5 µL; prod. no. qRnoCID0003485), nNOS/NOS1 (0.5 µL; prod. no. qRnoCED0009301), GLS (0.5 µL; prod. no. qRnoCID0007756), PRKAA1/AMPKα1 (0.5 µL; prod. no. qRnoCID0001262), PRKAA2/AMPKα2 (0.5 µL; prod. no. qRnoCID0006799), ESR1 (0.5 µL; prod. no. qRnoCID0009588), ESR2 (0.5 µL; prod. no. qRnoCID0008785), GPER (0.5 µL; prod. no. qRnoCED0007818), Ghrh-R (0.5 µL; prod. no. qRnoCED0003825, and GAPDH (0.5 µL; prod. no. qRnoCID0057018)], cDNA sample (2 µL), and iTaq^™^ Universal SYBR^®^ Green Supermix (5 µL, prod. no. 1725121; Bio-Rad). PCR samples were added to individual wells of hard-shell 384-well PCR plates (prod. no. HSP3805, Bio-Rad) for analysis in a CFX384^TM^ Touch Real-Time PCR Detection System (Bio-Rad) as follows: initial 30 sec 95 °C denaturation, followed by 40 cycles of (1) 3 sec incubation at 95 °C and (2) 45 sec incubation at 60 °C for GAD1/GAD_67_, ESR1; 60.5 °C for PRKAA1/AMPKα1, PRKAA2/AMPKα2; 59.9 °C for GAD2/GAD_65_, ESR2; 59.8 °C for GPER; 59.1 °C for SF-1/NR5A1; 58.8 °C for GLS; 58.5 °C for Ghrh; 58 °C for nNOS/NOS1; or 57.3 °C for GAPDH, respectively. Melt curve analyses were performed to detect nonspecific products and primer dimers. Data were analyzed by the comparative Ct (2^−ΔΔCt^) method (Livak & Schmittgen, 2001).

### Western Blot Analysis of VMNdm SF-1 Protein Content

For each animal, VMNdm tissue was bilaterally micropunch-dissected from 100 µm-thick frozen sections using calibrated hollow needle tools (Stoelting Co., Wood Dale, IL), and collected into lysis buffer (2% sodium dodecyl sulfate [SDS], 0.05 M dithiothreitol, 10% glycerol, 1 mM EDTA, 60 mM Tris-HCl, pH 7.2). For each treatment group, VMNdm tissue lysate aliquots from individual subjects were combined to create triplicate sample pools for SF-1 protein analysis. Sample pool proteins were separated by electrophoresis in Bio-Rad TGX 10% stain-free gels (prod. no. 1610183, Bio-Rad Laboratories Inc., Hercules, CA). Stain-Free imaging technology for total protein measurement was used as the loading control, as described (Bheemanapally et al., 2021; Briski et al., 2020; Ibrahim et al., 2020; Roy et al., 2023). After separation, gels were activated by UV light (1 min) in a Bio-Rad ChemiDoc MP Imaging System for quantification of individual lane total protein. Proteins were transferred to 0.45-µm PVDF-Plus membranes (prod. no. 121639; Data Support Co., Panorama City, CA), for FreedomRocker^™^ Blotbot^®^ (Next Advance, Inc., Troy, NY) automated wash and antibody incubation processing. Non-specific immunoreagent binding was abated by pretreatment blocking of membranes with Tris-buffer saline, pH 7.4, 10 mM tris hydrochloride, 50 mM sodium chloride (TBS) supplemented with 0.1% Tween-20 and 2% bovine serum albumin or SuperBlock^™^ Blocking Buffer (ThermoFisherSci., Waltham, MA). Membranes were incubated for 36–42 h, at 4 °C, with a rabbit primary polyclonal antiserum raised against SF-1 (prod. no. PA5-41967, 1:2000; Invitrogen, Waltham, MA). Membranes were next incubated with goat anti-rabbit horseradish peroxidase-labeled secondary antibodies (1:5000; prod. no. NEF812001EA; PerkinElmer, Waltham, MA), before exposure to maximum sensitivity SuperSignal WestFemto chemiluminescent substrate (prod. no. 34096; ThermoFisherSci.). The chemiluminescence optical density (O.D.) value measured for each target protein band was normalized to total protein in that lane using ChemiDoc MP Image Lab^™^ 6.0.0 software. Bio-Rad Stain-Free gels contain a proprietary trihalo compound that is directly incorporated into the gel chemistry; this compound lacks inherent fluorescence, but renders in-gel proteins fluorescent upon UV photoactivation and thus measurable by O.D. Software sums all individual protein optical densities in a single lane, and relates that total protein O.D. value to target protein O.D. in the same lane, thereby deriving a normalized O.D. value. Each Western blot analysis employed precision plus protein molecular weight dual color standards (prod. no. 161-0374, Bio-Rad). Our figures depict, as Y axis labels denote mean normalized O.D. measures. The formula used for normalization is the ratio of specific target protein O.D./total in-lane protein O.D.

### Circulating Glucose and Counterregulatory Hormone Profiles

Plasma glucose concentrations were measured in duplicate for each subject using an ACCU-CHECK Aviva-plus glucometer (Roche Diagnostic Corporation, Indianapolis, IN), as described (Napit et al., 2019). Circulating corticosterone (prod. no. ADI-900-097; Enzo Life Sciences, Inc., Farmingdale, NY), glucagon (prod. no. EZGLU-30K, EMD Millipore, Billerica, MA), and GH (prod. no. KRC5311; Invitrogen, Waltham, MA] concentrations were determined in duplicate using commercial ELISA kit reagents, as reported (Sapkota et al., 2023; Ibrahim et al., 2019).

### Statistics

Mean normalized mRNA, glucose, and counterregulatory hormone values were analyzed across treatment groups by two-way analysis of variance and Student Newman Keuls *post-hoc* test. Differences of *p* < 0.05 were considered significant. In each figure, statistical differences between specific pairs of treatment groups are denoted as follows: *p < 0.05; **p < 0.01; ***p < 0.001.

## Results

VMNdm Ghrh neurons express the transcription factor SF-1, which exerts well-documented regulatory effects on energy and glucose homeostasis. Current research employed siRNA reagents to repress VMN SF-1 gene expression *in vivo* to investigate the premise that in male rat Ghrh neurons, SF-1 controls eu- and/or hypoglycemia-associated expression patterns of gene transcripts that produce counterregulatory neurotransmitter biosynthetic pathway and ER variant proteins. Individual VMNdm Ghrh neurons were obtained by *in situ* gene immunocytochemistry/laser-catapult-microdissection methods for single-cell multiplex single-cell qPCR analyses. The correlated study hypothesis that VMN SF-1 gene expression is crucial for optimal eu- and/or hypoglycemic patterns of corticosterone, glucagon, and GH secretion in this sex was also be addressed.

**Figure 1.**
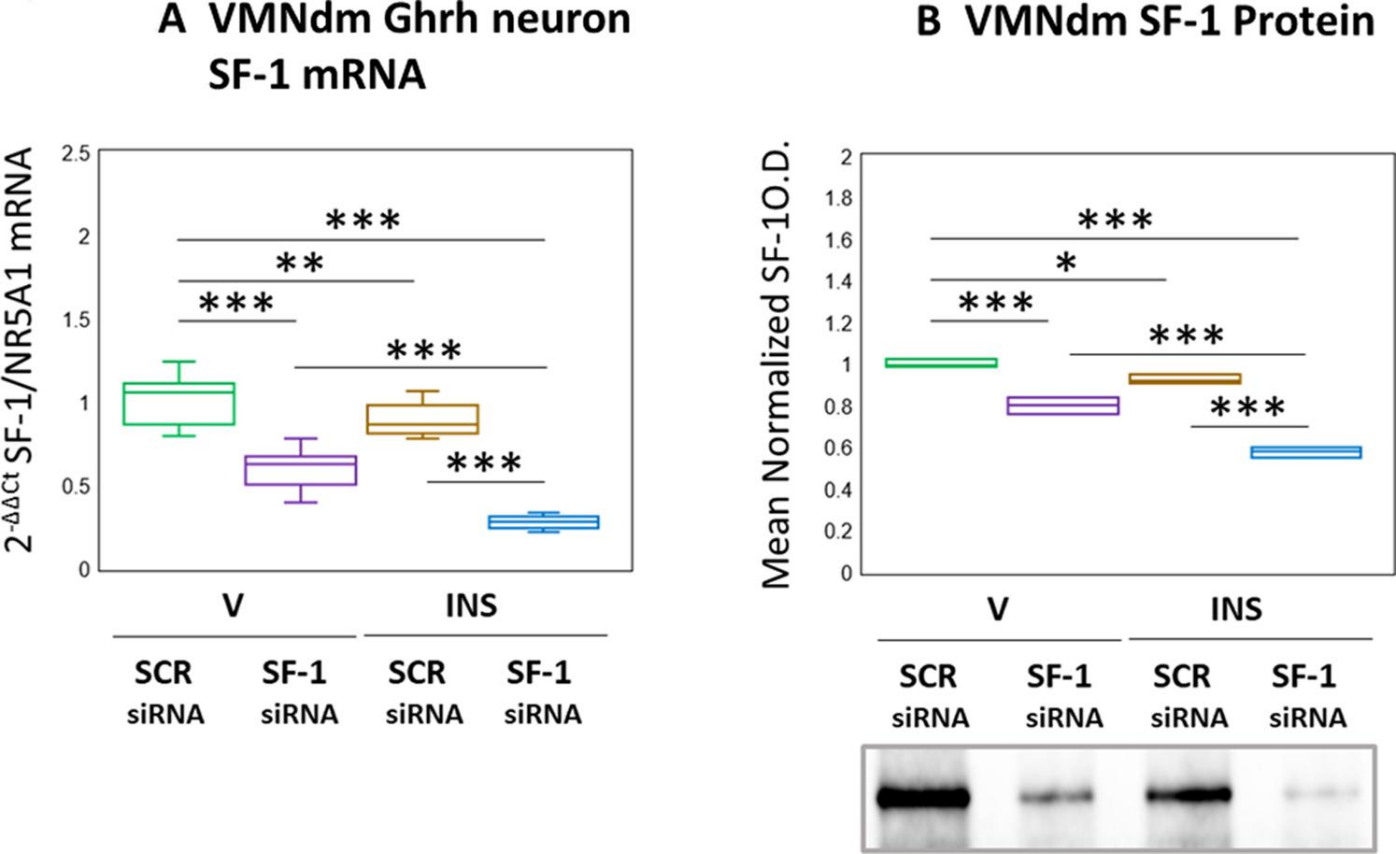
Effects of ventromedial hypothalamic nucleus steroidogenic factor-1 (SF-1) gene knockdown on dorsomedial VMN (VMNdm) growth hormone releasing hormone (Ghrh) neuron SF-1 mRNA and VMNdm SF-1 protein profiles in Eu- versus hypoglycemic male rats. Male rats were randomly assigned to treatment groups (*n* = 6/group) wherein animals were pretreated by bilateral intra-VMN administration of SF-1 or control/scramble (SCR) siRNA seven days prior to subcutaneous (sc) injection of vehicle (V) or neutral protamine hagedorn insulin (INS; 10.0 U/kg *bw*). Individual brains were into alternating 10 or 100 μm-thick fresh frozen sections for laser-catapult-microdissection of individual Ghrh-immunopositive neurons or micropunch dissection of VMNdm tissue, respectively. Figure A depicts results of single-cell qPCR SF-1 mRNA analysis. Data are presented in box-and-whisker plot format, which displays the median, lower and upper quartiles, and lower and upper extremes of a data set. Plots depict mean normalized VMNdm Ghrh neuron SF-1 transcript measures for the following treatment groups: SCR siRNA/V (green box-and-whisker plots, *n* = 12); SF-1 siRNA/V (purple box-and-whisker plots; *n* = 12); SCR siRNA/INS (golden box-and-whisker plots; *n* = 12; SF-1 siRNA/INS (blue box-and-whisker plots; male: *n* = 12). For each treatment group, aliquots of micropunched VMNdm tissue obtained from each animal were combined to create triplicate sample pools for Western blot analysis of SF-1 protein. Figure B depicts mean SF-1 protein optical density values for the treatment groups described above. mRNA and protein data were analyzed by two-way ANOVA and Student-Neuman-keuls *post-hoc* test, using GraphPad prism, vol. 8 software. Statistical differences between discrete pairs of treatment groups are denoted as follows: *p < 0.05; **p < 0.01; ***p < 0.001.

Results presented in Figure 1 depict effects of SF-1 gene silencing on eu- versus hypoglycemic patterns of SF-1 mRNA and protein expression in VMNdm Ghrh neurons. Data in Figure 1A show that SF-1 siRNA administration caused significant diminution of SF-1 transcript content of Ghrh nerve cells harvested from euglycemic animals (*F*_(3,44)_ = 117.45, *p* < 0.001; Pretreatment effect: *F*_(1.44)_ = 288.71, *p* < 0.001; Treatment effect: *F*_(1,44)_ = 53.04, *p* < 0.001; Pretreatment/treatment interaction: *F*_(1,44)_ = 10.59, *p* = 0.002). SF-1 gene expression in this nerve cell type was reduced in response to hypoglycemia; INS-injected animals exhibited further reductions in this mRNA profile as a consequence of SF-1 siRNA pretreatment. As seen in Figure 1B, SF-1 gene silencing was effective in reducing mean normalized SF-1 protein content of microdissected VMNdm tissue from V- or INS-injected rats by (*F*_(3,8)_ = 129.19, *p* < 0.001; Pretreatment effect: *F*_(1.8)_ = 283.47, *p* < 0.001; Treatment effect: *F*_(1,8)_ = 8379, *p* < 0.001; Pretreatment/treatment interaction: *F*_(1,8)_ = 20.31, *p* = 0.002). Data show that net tissue SF-1 protein in this location was refractory to hypoglycemia.

**Figure 2.**
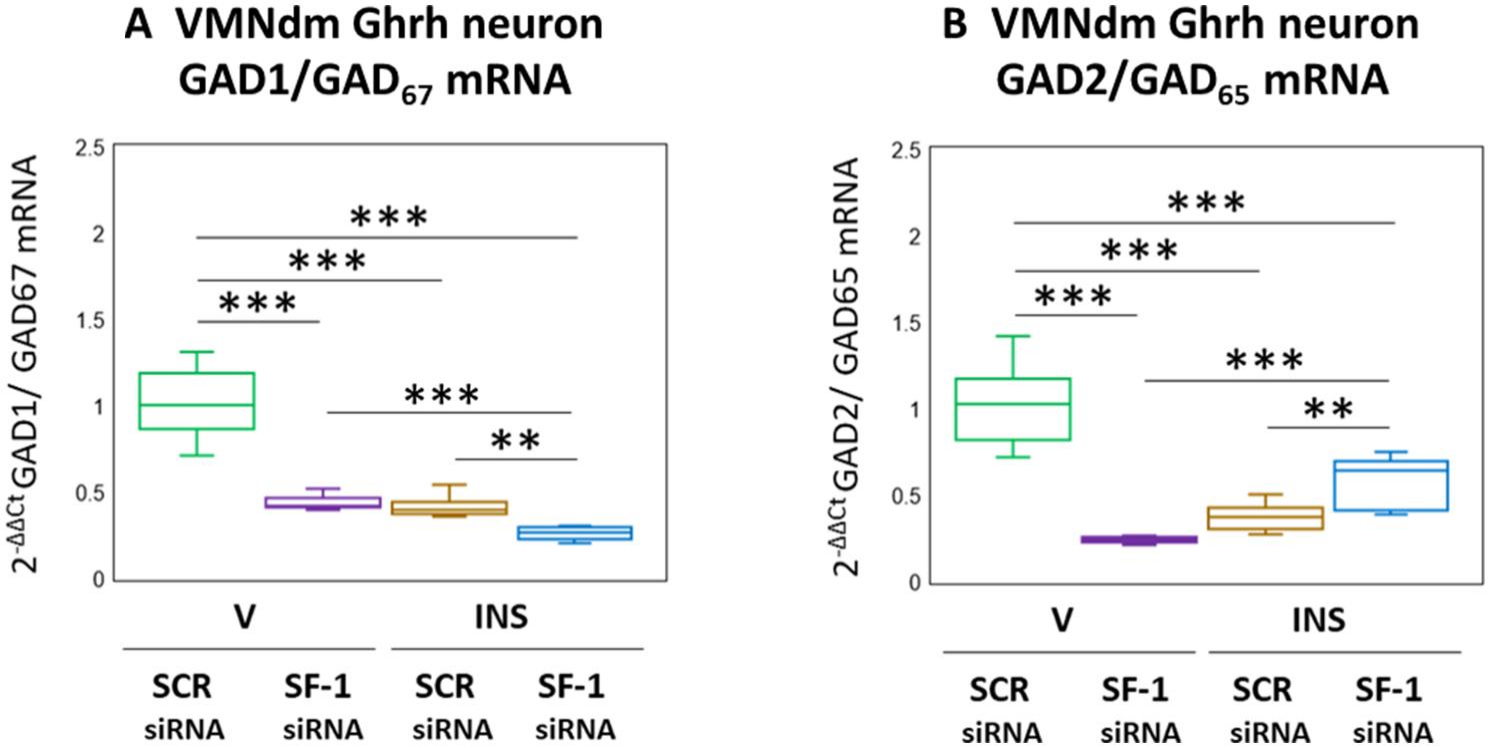
Effects of VMN SF-1 gene knockdown on VMNdm ghrh neuron glutamate decarboxylase (GAD)-1/GAD_67_ and GAD2/GAD_65_ gene transcription in V- or INS-injected male rats. Results present mean normalized GAD1 (A) and GAD2 (B) mRNA profiles for VMNdm ghrh neurons acquired from male rats treated as follows: SCR siRNA/V (green box-and-whisker plots; *n* = 12); SF-1 siRNA/V (purple box-and-whisker plots; *n* = 12); SCR siRNA/INS (golden box-and-whisker plots; male: *n* = 12); SF-1 siRNA/INS (blue box-and-whisker plots; *n* = 12). Normalized mRNA data were analyzed by two-way ANOVA and Student-Neuman-keuls *post-hoc* test, using GraphPad prism, vol. 8 software. Statistical differences between discrete pairs of treatment groups are denoted as follows: *p < 0.05; **p < 0.01; ***p < 0.001.

The amino acid transmitter GABA suppresses counterregulatory hormone release. The enzyme GAD catalyzes rate-limiting production of GABA from glutamate, and occurs in the brain as 67 (GAD1/GAD_67_) and 65 (GAD2/GAD_65_) kDa molecular weight isoforms differ with respect to amino acid primary structure, nerve cell subcellular localization, and regulation [Behar, 2009]. The distinctive gene transcripts that encode these GAD variants are co-expressed in VMNdm Ghrh neurons. Data presented in Figure 2A,B depicts the effects of SF-1 siRNA patterns of Ghrh neuron GAD1 and GAD2 gene expression under conditions of eu- versus hypoglycemia. As shown in Figure 2A, V-injected rats exhibited significant SF-1 siRNA-associated reductions in Ghrh nerve cell GAD1 mRNA (*F*_(3,44)_ = 121.37, *p* < 0.001; Pretreatment effect: *F*_(1.44)_ = 146.43, *p* < 0.001; Treatment effect: *F*_(1,44)_ = 166.90, *p* < 0.001; Pretreatment/treatment interaction: *F*_(1,44)_ = 50.78, *p* < 0.001). Hypoglycemia suppressed GAD1 gene expression in this neuron population; this inhibitory response was exacerbated by SF-1 siRNA pretreatment. Outcomes shown in Figure 2B illustrate SF-1 siRNA-mediated suppression of baseline GAD2 transcription (*F*_(3,44)_ = 75.73, *p* < 0.001; Pretreatment effect: *F*_(1.44)_ = 51.98, *p* < 0.001; Treatment effect: *F*_(1,44)_ = 17.51, *p* < 0.001; Pretreatment/treatment interaction: *F*_(1,44)_ = 157.70, *p* < 0.001). Inhibitory effects of hypoglycemia on this gene profile were partially reversed by SF-1 siRNA pretreatment.

**Figure 3.**
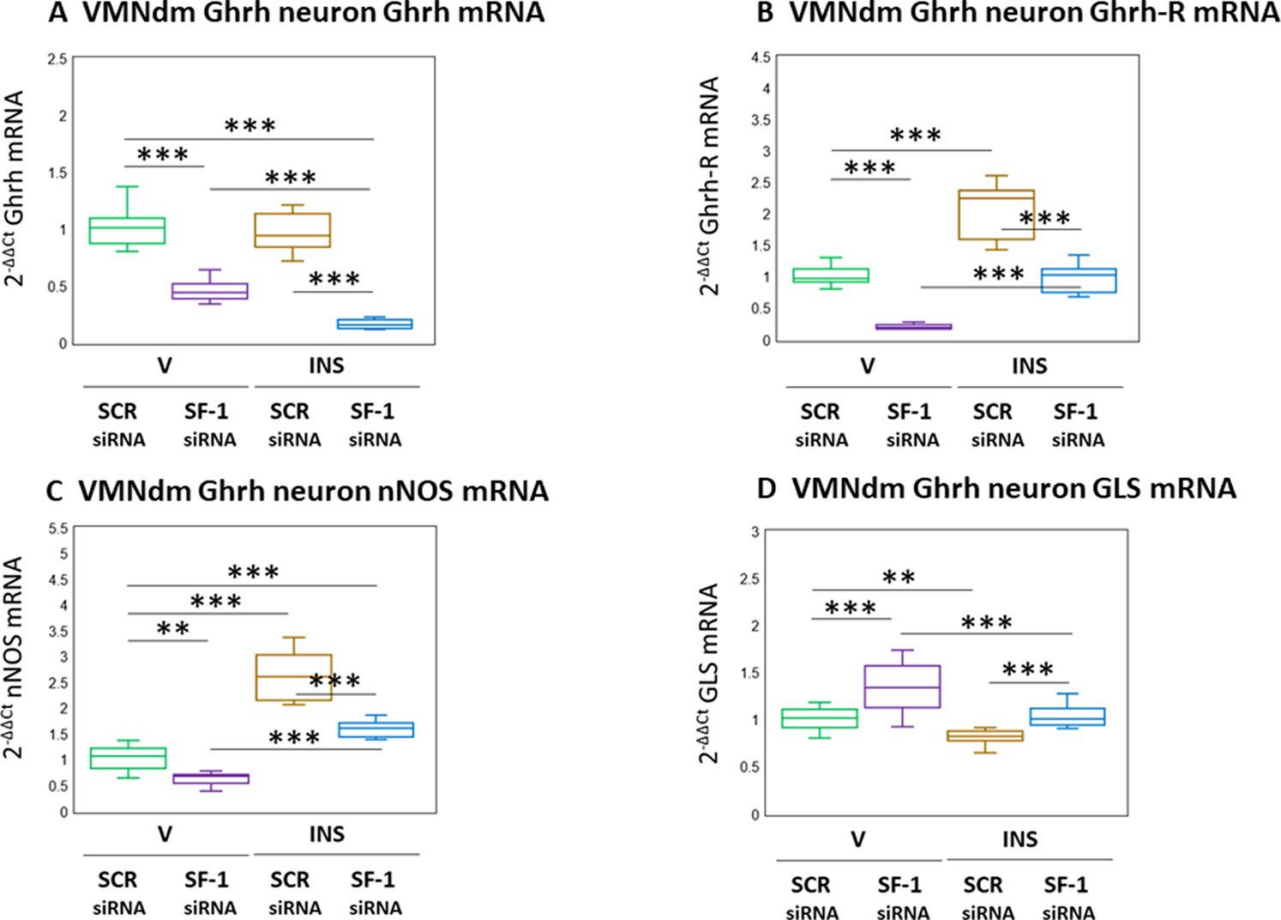
Effects of SF-1 gene silencing on hypoglycemic patterns of Ghrh, Ghrh receptor (Ghrh-R), nitric oxide synthase (nNOS), and glutaminase (GLS) mRNA expression in male rat VMNdm Ghrh neurons. Data depict mean normalized Ghrh (A), Ghrh-R (B), nNOS (C), and GLS (D) mRNA measures for VMNdm Ghrh neurons collected following SCR siRNA/V (*n* = 12); SF-1 siRNA/V (*n* = 12); SCR siRNA/INS (*n* = 12); or SF-1 siRNA/INS (*n* = 12) treatment. Normalized mRNA data were analyzed by two-way ANOVA and Student-Neuman-keuls *post-hoc* test, using GraphPad prism, vol. 8 software. Statistical differences between discrete pairs of treatment groups are denoted as follows: *p < 0.05; **p < 0.01; ***p < 0.001.

The neuropeptide Ghrh, lipid-permeable gas NO, and amino acid glutamate each have documented stimulatory effects on counterregulatory outflow, and are co-expressed in VMNdm Ghrh neurons. Figure 3 illustrates the effects of SF-1 gene knockdown on genes that encode Ghrh (Figure 3A) or the transmitter biosynthetic enzyme markers nNOS (Figure 3C) and GLS (Figure 3D). Figure 3A illustrates suppressive effects of SF-1 mRNA on patterns of Ghrh gene expression in V- or INS-injected animals (*F*_(3,44)_ = 128.90, *p* < 0.001; Pretreatment effect: *F*_(1.44)_ = 353.05, *p* < 0.001; Treatment effect: *F*_(1,44)_ = 21.72, *p* < 0.001; Pretreatment/treatment interaction: *F*_(1,44)_ = 11.93, *p* < 0.001). Previous studies showed that Ghrh modulates expression of mRNAs for Ghrh nerve cell co-expressed neurotransmitters; documentation that these neurons contain Ghrh-R transcripts infers that this neuropeptide may control those gene profiles by direct action on Ghrh neurons. As seen in Figure 3B, baseline Ghrh nerve cell Ghrh-R mRNA levels were decreased by SF-1 siRNA. Augmentation of these transcripts by hypoglycemia was averted by SF-1 gene knockdown (*F*_(3,44)_ = 115.62, *p* < 0.001; Pretreatment effect: *F*_(1.44)_ = 174.34, *p* < 0.001; Treatment effect: *F*_(1,44)_ = 168.55, *p* < 0.001; Pretreatment/treatment interaction: *F*_(1,44)_ = 3.97, *p* = 0.053). Data presented in Figure 3C show that Ghrh neuron nNOS mRNA levels were decreased by SF-1 gene silencing (*F*_(3,44)_ = 113.26, *p* < 0.001; Pretreatment effect: *F*_(1.44)_ = 76.75, *p* < 0.001; Treatment effect: *F*_(1,44)_ = 247.88, *p* < 0.001; Pretreatment/treatment interaction: *F*_(1,44)_ = 15.14, *p* < 0.001). Stimulation of nNOS transcript levels by hypoglycemia was attenuated by SF-1 siRNA pretreatment. Ghrh nerve cell GLS transcripts were up-regulated by SF-1 mRNA (Figure 3D; *F*_(3,44)_ = 21.21, *p* < 0.001; Pretreatment effect: *F*_(1.44)_ = 35.25, *p* < 0.001; Treatment effect: *F*_(1,44)_ = 27.08, *p* < 0.001; Pretreatment/treatment interaction: *F*_(1,44)_ = 1.31, *p* = 0.258). Hypglycemic inhibition of this gene profile was reversed by SF-1 siRNA pretreatment.

**Figure 4.**
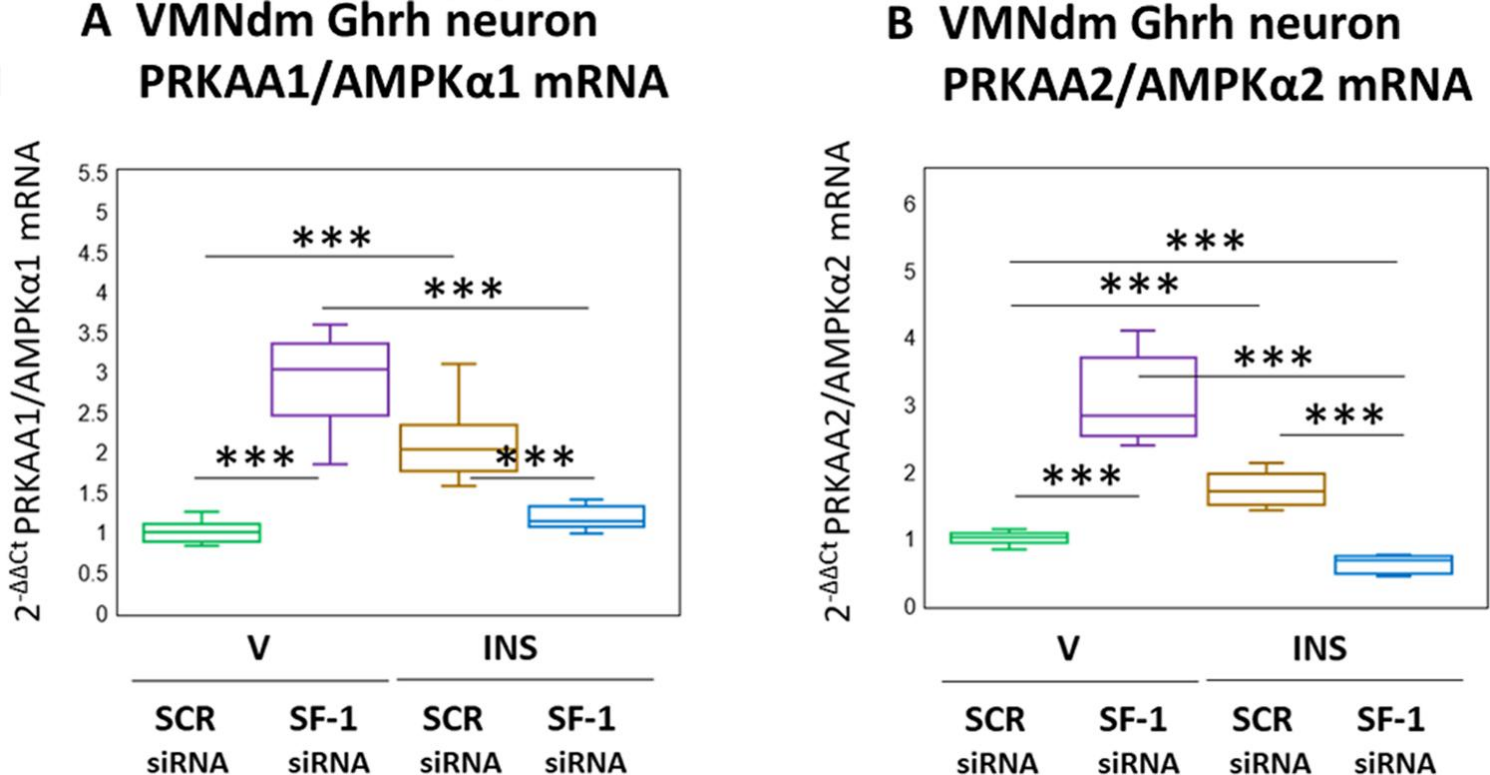
SF-1 gene knockdown effects on VMNdm ghrh nerve cell PRKAA1/AMPKα1 and PRKAA2/AMPKα2 gene expression in Eu- versus hypoglycemic male rats. Data depict mean normalized PRKAA1/AMPKα1 (A) and PRKAA2/AMPKα2 (B) mRNA values for VMNdm ghrh neurons collected after SCR siRNA/V (*n* = 12); SF-1 siRNA/V (*n* = 12); SCR siRNA/INS (*n* = 12); or SF-1 siRNA/INS (*n* = 12) treatment. Normalized mRNA data were analyzed by two-way ANOVA and Student-Neuman-keuls *post-hoc* test, using GraphPad prism, vol. 8 software. Statistical differences between discrete pairs of treatment groups are denoted as follows: *p < 0.05; **p < 0.01; ***p < 0.001.

Results presented in Figure 4 depict the effects of SF-1 gene knockdown on VMNdm Ghrh nerve cell AMPKα1 and AMPKα2 gene expression. As shown in Figure 4A, AMPKα1 mRNA content was significantly increased in response to SF-1 siRNA (*F*_(3,44)_ = 67.23, *p* < 0.001; Pretreatment effect: *F*_(1.44)_ = 21.26, *p* < 0.001; Treatment effect: *F*_(1,44)_ = 8.89, *p* = 0.005; Pretreatment/treatment interaction: *F*_(1,44)_ = 171.53, *p* < 0.001). Stimulatory effects of hypoglycemia on this gene profile were reversed by SF-1 siRNA pretreatment. Likewise, AMPKα2 transcripts (Figure 4B) were respectively increased or decreased by SF-1 knockdown under eu- versus hypoglycemic conditions (*F*_(3,44)_ = 114.53, *p* < 0.001; Pretreatment effect: *F*_(1.44)_ = 21.91, *p* < 0.001; Treatment effect: *F*_(1,44)_ = 73.37, *p* < 0.001; Pretreatment/treatment interaction: *F*_(1,44)_ = 249.31, *p* < 0.001).

**Figure 5.**
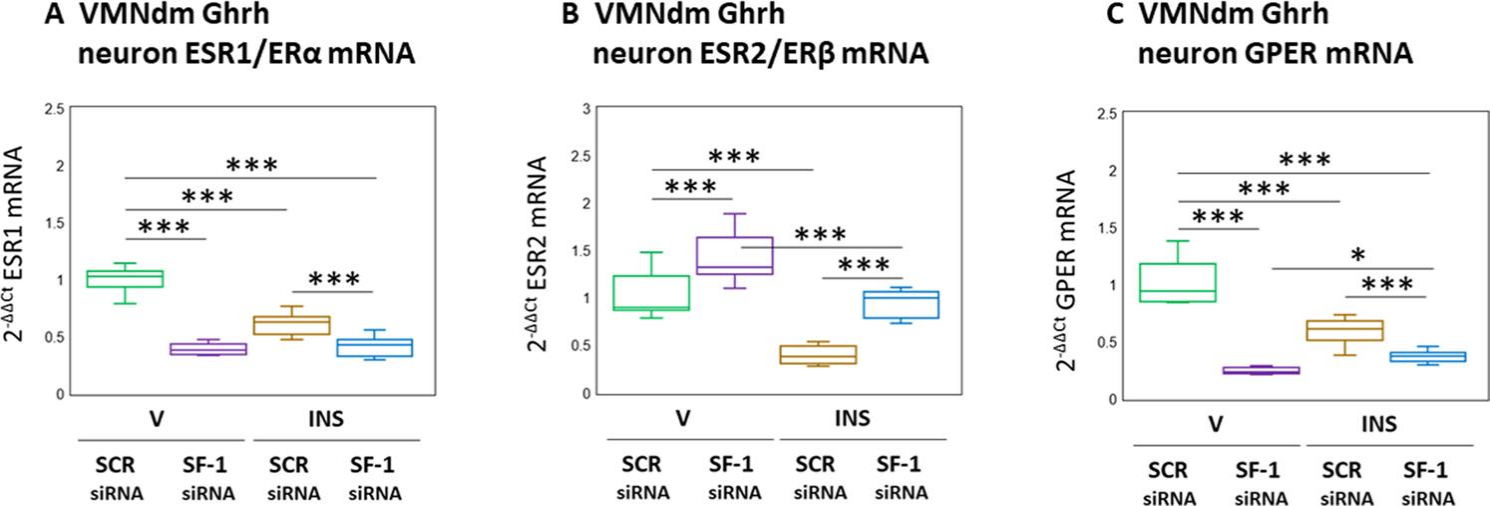
Patterns of VMNdm ghrh neuron Estrogen receptor-alpha (ESR1/ERα), ER-Beta (ESR2/ERβ), and G protein-Coupled membrane Estrogen receptor-1 (GPER) gene expression in SF-1 siRNA-pretreated V- or INS-injected male rats. Data depict mean normalized ESR1 (A), ESR2 (B), and GPER (C) gene expression in VMNdm ghrh neurons after the following combinatory treatments: SCR siRNA/V (*n* = 12); SF-1 siRNA/V (*n* = 12); SCR siRNA/INS (*n* = 16); SF-1 siRNA/INS (*n* = 16). Normalized mRNA data were analyzed by two-way ANOVA and Student-Neuman-keuls *post-hoc* test, using GraphPad prism, vol. 8 software. Statistical differences between discrete pairs of treatment groups are denoted as follows: *p < 0.05; **p < 0.01; ***p < 0.001.

Figure 5 depicts expression patterns of nuclear [ESR1/ERα (Figure 5A); ESR1/ERβ (Figure 5B)] and membrane (Figure 5C) ER mRNAs in male rat VMNdm Ghrh neurons. Data show that disclose that baseline ESR1 (*F*_(3,44)_ = 137.53, *p* < 0.001; Pretreatment effect: *F*_(1.44)_ = 278.20, *p* < 0.001; Treatment effect: *F*_(1,44)_ = 57.34, *p* < 0.001; Pretreatment/treatment interaction: *F*_(1,44)_ = 77.05, *p* < 0.001) and GPER (*F*_(3,44)_ = 105.24, *p* < 0.001; Pretreatment effect: *F*_(1.44)_ = 225.46, *p* < 0.001; Treatment effect: *F*_(1,44)_ = 21.46, *p* < 0.001; Pretreatment/treatment interaction: *F*_(1,44)_ = 68.79, *p* < 0.001) transcripts were significantly decreased, whereas as ESR2 (*F*_(3,44)_ = 57.89, *p* < 0.001; Pretreatment effect: *F*_(1.44)_ = 71.22, *p* < 0.001; Treatment effect: *F*_(1,44)_ = 100.26, *p* < 0.001; Pretreatment/treatment interaction: *F*_(1,44)_ = 2.21, *p* = 0.145) was augmented following SF-1 gene silencing. Hypoglycemia suppressed expression of these ER variant mRNAs in VMNdm Ghrh neurons; SF-1 siRNA pretreatment amplified this decline in ESR1 and GPER transcripts, but reversed this inhibitory ESR2 transcriptional response.

**Figure 6.**
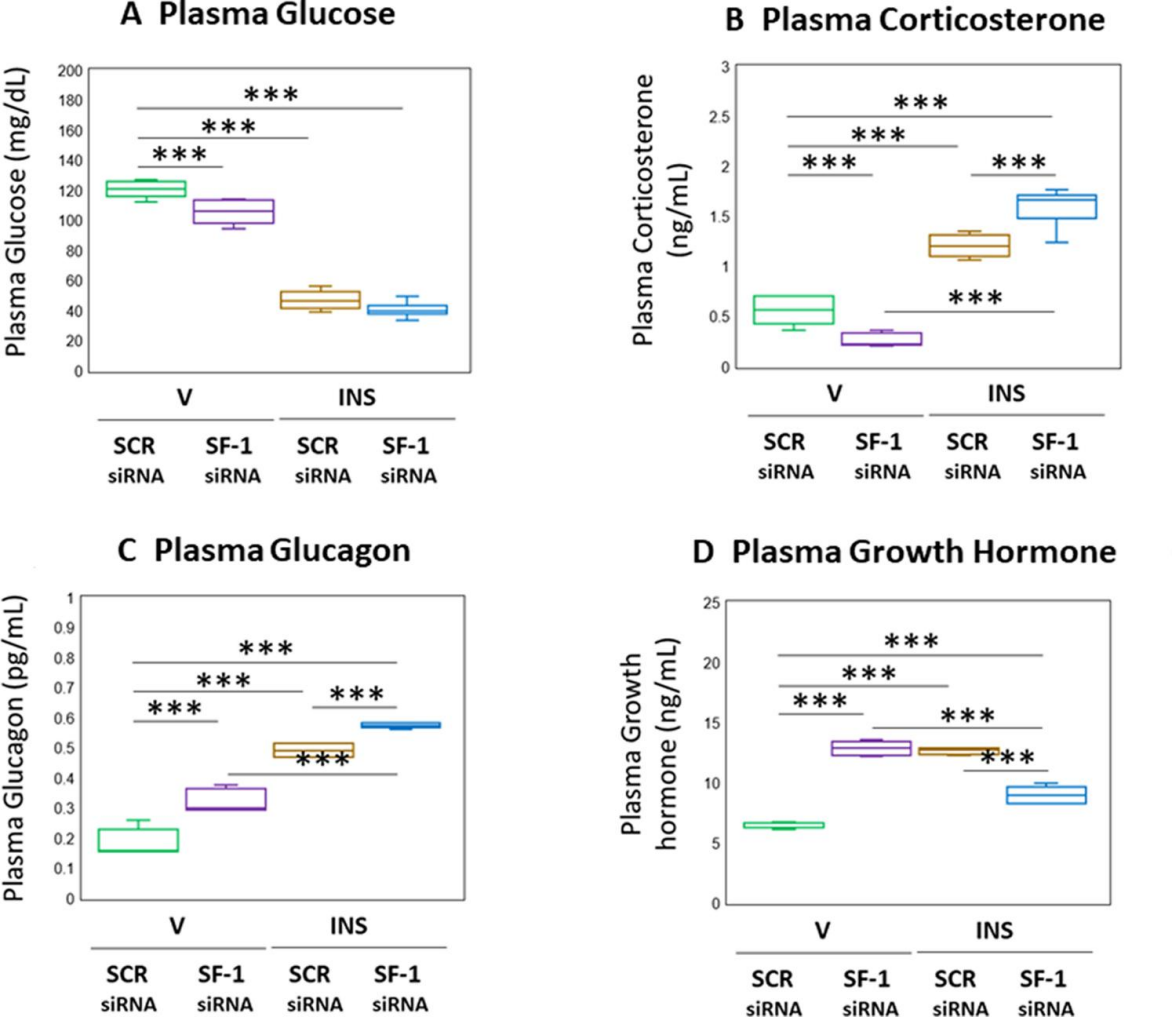
Effects of VMN SF-1 gene knockdown on plasma glucose and Counter-Regulatory hormone profiles in Eu- or hypoglycemic male rats. Plasma samples were obtained from groups of SF-1 or SCR siRNA-pretreated male rats one hour after *sc* injection of V or INS, and analyzed for glucose (A), corticosterone (B), glucagon (C), or growth hormone (D) concentrations. In each panel, individual treatment group data depict mean plasma concentrations ± S.E.M. for *n* = 6 samples. Data were analyzed by two-way ANOVA and Student-Neuman-keuls *post-hoc* test, using GraphPad prism, vol. 8 software. *p < 0.05, **p < 0.01, ***p < 0.001.

Figure 6 depicts the effects of VMN SF-1 gene knockdown on circulating glucose and counterregulatory hormone concentrations in eu- and hypoglycemic male rats. As seen in Figure 6A, euglycemic animals exhibited a reduction in plasma glucose levels in response to SF-1 silencing (*F*_(3,20)_ = 252.40, *p* < 0.001; Pretreatment effect: *F*_(1.20)_ = 17.70, *p* < 0.001; Treatment effect: *F*_(1,20)_ = 736.77, *p* < 0.001; Pretreatment/treatment interaction: *F*_(1,20)_ = 2.76, *p* = 0.112). The magnitude of hypoglycemia at +1 hour post-INS injection was not different between SF-1 versus SCR-pretreated rats. Corticosterone concentrations were decreased in SF-1 siRNA-pretreated, V-injected rats (Figure 6B; *F*_(3,20)_ = 125.76, *p* < 0.001; Pretreatment effect: *F*_(1.20)_ = 0.81, *p* = 0.378; Treatment effect: *F*_(1,20)_ = 335.64, *p* < 0.001; Pretreatment/treatment interaction: *F*_(1,20)_ = 40.82, *p* < 0.001). Hypoglycemic hypercorticosteronemia was amplified by SF-1 siRNA pretreatment. Data in Figure 6C show that basal and hypoglycemic patterns of glucagon secretion were each up-regulated in response to SF-1 gene knockdown (*F*_(3,20)_ = 179.08, *p* < 0.001; Pretreatment effect: *F*_(1.20)_ = 70.49, *p* < 0.001; Treatment effect: *F*_(1,20)_ = 462.80, *p* < 0.001; Pretreatment/treatment interaction: *F*_(1,20)_ = 3.95, *p* < 0.001). Circulating GH concentrations were elevated in euglycemic rats by SF-1 gene knockdown (Figure 6D; *F*_(3,20)_ = 114.53, *p* < 0.001; Pretreatment effect: *F*_(1.20)_ = 21.91, *p* < 0.001; Treatment effect: *F*_(1,20)_ = 73.37, *p* < 0.001; Pretreatment/treatment interaction: *F*_(1,20)_ = 249.31, *p* < 0.001). Hypoglycemic stimulation of GH secretion was reversed by SF-1 siRNA pretreatment.

## Discussion

The transcription factor SF-1, uniquely expressed in the VMN, acts to regulate systemic glucostasis. VMNdm Ghrh neurons express SF-1 mRNA (Sapkota et al., 2023). Here, *in vivo* gene silencing tools were used together with single-cell multiplex qPCR and ELISA methods to address the premise that SF-1 may govern Ghrh nerve cell counterregulatory neurotransmission and counterregulatory hormone secretion in the male rat. Study outcomes affirm SF-1 control of basal and hypoglycemic expression patterns of mRNAs that encode enzyme markers for NO, glutamate, and GABA. This transcription factor also shapes Ghrh neuron receptivity to Ghrh and estradiol. SF-1 up-regulates baseline ERα, ERβ, and GPER gene expression in this cell type, but elicits distinctive adjustments in ER variant transcription during hypoglycemia. Results provide unique evidence that SF-1 governance of AMPKα isoform gene profiles shift from inhibitory-to-stimulatory during glucostasis versus imbalance. Data moreover substantiate VMN SF-1 regulation of circulating corticosterone, glucagon, and GH concentrations during eu- and hypoglycemia. Further research is warranted to establish whether, how, and where distinctive SF-1 – sensitive neurotransmitters characterized here may communicate sequelae of SF-1 transcriptional regulation to the neural glucostatic network. There also remains a need for insight on if and how signaling by individual ER variants may affect SF-1 control of Ghrh counterregulatory neurotransmission.

The overarching goal of the current research was to obtain proof-of-concept that genetic manipulation of VMN SF-1 gene expression affects VMNdm Ghrh neuron estrogen receptor variant, energy sensor, and/or transmitter marker protein gene expression. Given the considerable quantity of work required to achieve this goal, we chose to begin by using a single sex, the male, as the experimental subject as this sex has been the default choice over decades of neuroscience research. Present outcomes provide solid justification for the expansion of this line of research through the application of the current experimental design in studies on female.

Present data corroborate earlier reports that Ghrh neurons are characterized by the co-presence of SF-1 transcripts with mRNAs that produce chemically-diverse transmitters of documented impact on counterregulation, i.e., Ghrh, NO, glutamate, and GABA) (Sapkota et al., 2023). Findings here extend those observations with novel proof that SF-1 regulates basal and hypoglycemic expression patterns of these genes. Outcomes validate the efficacy of the present gene knockdown treatment paradigm, involving bilateral administration of gene-targeted or SCR siRNA to the VMN, for down-regulation of SF-1 gene and protein expression in VMNdm Ghrh neurons. The issue of whether additional neurotransmitter nerve populations in that discrete nuclear subdivision express SF-1 remains unanswered. It is noted that quantifiable experimental stimulus-induced changes in SF-1 mRNA profiles, such as hypoglycemia-associated decrements in SF-1 gene expression observed here, do not provide confirmation that Ghrh SF-1 protein undergoes changes of parallel magnitude. Hence, the possibility that SF-1 protein expression in this cell type may be refractory to hypoglycemia, despite decreased SF-1 transcription, should not be discounted. Observations here of diminished Ghrh nerve cell Ghrh mRNA profiles in SF-1 siRNA-pretreated, V- or INS-injected rats relative to SCR-pretreated controls infer that Ghrh neuropeptide signaling is up-regulated by this transcription factor. Current data expand upon recent evidence that VMNdm Ghrh neurons express Ghrh-R gene transcripts with verification that this mRNA profile is augmented by SF-1 during eu- and hypoglycemia, and that this transcription factor may be a primary factor in hypoglycemic amplification of Ghrh-R gene expression.

In the brain, the conversion of glutamate to GABA is catalyzed by the biosynthetic enzyme GAD, which occurs as 67 kDa (GAD1/GAD_67_) and 65 kDa (GAD2/GAD_65_) kDa size variants encoded by distinct genes. Prior and current data affirm that GAD1 and GAD2 transcripts are co-expressed in VMNdm Ghrh neurons. These molecular weight variants differ with regard to amino acid primary structure, neuronal subcellular distribution, and regulation. GAD1/GAD_67_ expression is subject to transcriptional and posttranscriptional control, whereas GAD2/GAD_65_ is controlled by transcriptional and kinetic mechanisms (Behar, 2009). Localization of GAD2/GAD_65_ protein to axon terminals and vesicles versus intra-cytoplasmic GAD1/GAD_67_ infers the existence of distinctive vesicular versus cytoplasmic GABA pools and a potential role for GAD2/GAD_65_ in neurotransmission compared to GAD1/GAD_67_ involvement in cellular metabolic functions (Martin & Barke, 1998; Schousboe & Waagepetersen, 2017; Tavazzani et al., 2014). Current data show that SF-1 stimulates baseline expression of both GAD1 and-2 transcripts during euglycemia, yet imposes divergent control of GAD isoform mRNAs during hypoglycemia as SF-1 counteracts hypoglycemic inhibition of GAD1 transcription, yet is critical for down-regulated GAD2 gene profiles. SF-1 may thus be capable of discriminative control of vesicular versus cytoplasmic GABA production and activity in this nerve cell type during hypoglycemia; data suggest that SF-1 may inhibit the former pool while stimulating the latter. The direction of SF-1 regulation of GAD2 transcription profiles is evidently dependent upon glucose status, as this control shifts from stimulatory-to-inhibitory during eu- versus hypoglycemia; elucidation of the mechanisms that mediate this directional switch will require further investigation.

Previous studies showed that VMNdm Ghrh neurons express a critical enzyme component (i.e., GLS) of the biosynthetic pathway that manufactures the counterregulation-enhancing amino acid neurotransmitter glutamate (Sapkota et al., 2023). New results described here reveal that SF-1 suppresses GLS gene expression during eu- or hypoglycemia, and support the view that this transcription factor may mediate hypoglycemic inhibition of this gene profile. Ghrh neurons also release the diffusible counterregulation-stimulating free radical NO, which shows that this cell type engages in both non-receptor- and receptor-mediated signaling to elevate circulating glucose levels. Data here show that SF-1 gene silencing down-regulated basal and hypoglycemic patterns of nNOS gene expression, denoting a positive impact of this transcription factor on NO release. Thus, SF-1 evidently imposes the opposite, i.e., inhibitory versus stimulatory effects on counterrregulatory-enhancing neurochemicals glutamine and NO produced by Ghrh neurons. Outstanding issues that remain to be addressed include identification of cellular targets and functional consequences of SF-1 – dependent release of glutamate versus NO from VMNdm Ghrh neurons for counterregulatory outflow.

Current studies provide unique proof that hypoglycemia-sensitive AMPKα1 and AMPKα2 gene transcripts are co-expressed in VMNdm Ghrh neurons. The presence of molecular machinery for energy screening infers that this nerve cell type likely utilizes intrinsic metabolic cues to shape neurochemical signaling. It remains to be determined if AMPK activity state controls production and release of all or a subset of co-expressed transmitters. Data disclose that SF-1 inhibits expression of both AMPKα variant mRNAs in Ghrh neurons during euglycemia, yet is paradoxically crucial for hypoglycemic up-regulation of these gene profiles. Further research is warranted to characterize mechanisms that achieve this evident glucose-dependent shift from SF-1 inhibitory-to-stimulatory control of AMPKα mRNA expression. While current outcomes support the view that SF-1 may control adaptation of Ghrh nerve cell energy sensory function to glucose imbalance, it should be noted that effects of SF-1 knockdown alone or in combination with hypoglycemia on phosphorylation state of either AMPKα variant were not evaluated here. Thus, insight on the direction and magnitude of SF-1 regulatory impact on AMPK activity during eu- or hypoglycemia will require additional effort.

Project results show that SF-1 up-regulates expression of nuclear and membrane ER variant genes in VMNdm Ghrh/SF-1 neurons, inferring that this transcription factor may exert a net positive impact on cellular receptivity to estradiol. It is noted that measured adjustments in ER variant mRNA levels should not be viewed as definitive proof of corresponding changes in receptor protein production. This notion will remain speculative until analytical methods of requisite sensitivity for quantification of these proteins in single-cell samples become available. Outcomes reported here confirm that Ghrh neurons acquired from male rats exhibit down-regulated ERα, ERβ, and GPER gene profiles during hypoglycemia (Sapkota et al., 2023). While SF-1 evidently acts to antagonize hypoglycemic down-regulation of ERα and GPER gene expression, this transcription factor may coincidently inhibit ERβ transcription. The mechanisms that underlie this glucose-dependent switch from stimulatory to inhibitory SF-1 control of Ghrh nerve cell ERβ gene expression are not known. Additional research is also warranted to characterize the regulatory stimuli that impose inhibitory effects of hypoglycemia on Ghrh neuron ERα and GPER mRNA content. There is, above all, an urgent need to understand if SF-1 – dependent changes in ER variant-mediated estradiol signaling to Ghrh neurons during hypoglycemia are involved in neurotransmitter protein marker gene responses to glucose imbalance.

Application of *in vivo* SF-1 gene knockdown tools confirms a role for this VMN transcription factor in counterregulatory hormone release during hypoglycemia (Choi et al., 2013; Kim et al., 2012; Tong et al., 2007 2013). Data presented here show that SF-1 imposes distinctive regulatory effects on basal circulating counterregulatory hormone profiles, as SF-1 siRNA reduced circulating corticosterone levels, yet elevated glucagon and GH concentrations. The direction of SF-1 control of corticosterone is seemingly glucose-dependent as hypoglycemic hypercorticosteronemia was seen to be enhanced by SF-1 gene silencing. An inverse scenario concerns SF-1 regulation of GH secretion; while SF-1 acts to inhibit GH secretion during euglycemia, this transcription factor evidently mediates hypoglycemic stimulation of this hormone profile. On the other hand, SF-1 has a consistently negative impact on glucagon release irrespective of systemic glucose status. Further research is required to identify the Ghrh nerve cell neurotransmitter(s) that impose SF-1-dependent control of individual counterregulatory hormones. Outcomes show that SF-1 gene knockdown did not modify plasma glucose levels after INS injection, despite measurable effects on circulating corticosterone, glucagon, and GH. As glucose measures were acquired at a single time point following INS treatment, the prospect that siRNA treatment might have affected glycemic profiles over some time interval prior to sacrifice cannot be discounted. It is reasonable to presume that circulating glucose concentrations may undergo dynamic change due to VMN SF-1 governance of counter-regulatory hormone release, or control of neural mechanisms governing hepatic gluconeogenic or glycogenolytic functions. Current results may thus provide a snapshot of a temporal phase during which plasma glucose levels are normalized after INS administration as an adaptive reaction to SF-1 – dependent actions that control contra-regulatory outflow.

Summary of project outcomes: Present data support the likely function of VMNdm Ghrh neurons as a cellular effector of SF-1 regulation of systemic glucostasis in the male rat. SF-1 regulates expression patterns of genes that encode the neuropeptide transmitter Ghrh as well as co-expressed counterregulation-inhibiting or -enhancing neurochemicals of distinctive chemical structure and spatial/temporal release profiles; these outcomes infer that SF-1 may coordinate complex, multi-modal neurotransmitter input to the brain glucostatic circuitry. Continuing research efforts intend to address the issue of whether such control involves AMPK- and/or ER-dependent mechanisms. Results provide novel evidence for SF-1 regulation of basal counterregulatory hormone secretion profiles in the male, documenting stimulation of corticosterone versus inhibition of glucagon and GH. This transcription factor also imposes distinctive control of these hormone profiles during hypoglycemia. Current research supports the need to characterize downstream cell targets of SF-1 – dependent neurotransmitter signaling and functional impact of this input on systems-level neural regulation of glucostasis.

## CRediT Authorship Statement

Subash Sapkota: conceptualization, investigation, formal analysis, validation, data curation, visualization, writing – original draft, writing – review and editing, visualization; Sagor C. Roy: investigation, formal analysis, validation, data curation; Rami Shrestha: investigation, formal analysis, validation, data curation; Karen P. Briski: conceptualization, writing – original draft, writing – review and editing, supervision, project administration, funding acquisition

## Ethical Approval

All animal experimental was carried out in compliance with the National Institutes of Health Guide for the Care and Use of Laboratory Animals, 8th Edition, as stated in the submitted manuscript. Sex of animals used is included, along with discussion of sex impacts on study outcomes.

## Data Availability

The data that support the findings of this study are available from the corresponding author upon reasonable request.

## Abbreviations

ERα: estrogen receptor-alpha
ERβ: estrogen receptor-beta
GAD1/GAD_67_: glutamine decarboxylase (GAD)_67_
GAD2/GAD_65_: GAD_65_
GH: growth hormone
Ghrh: growth hormone-releasing hormone
Ghrh-R: Ghrh receptor
GLS: glutaminase
GPER: G protein-coupled estrogen receptor-1
IIH: insulin-induced hypoglycemia
INS: insulin
nNOS: neuronal nitric oxide synthase
NO: nitric oxide
*sc*: subcutaneous
SF-1/Nr5a1: steroidogenic factor-1
VMN: ventromedial hypothalamic nucleus
VMNdm: VMN dorsomedial subdivision
